# How does narrative medicine impact medical trainees’ learning of professionalism? A qualitative study

**DOI:** 10.1186/s12909-021-02823-4

**Published:** 2021-07-21

**Authors:** Chien-Da Huang, Chang-Chyi Jenq, Kuo-Chen Liao, Shu-Chung Lii, Chi-Hsien Huang, Tsai-Yu Wang

**Affiliations:** 1 Chang Gung Medical Education Research Centre, Chang Gung Memorial Hospital, Chang Gung University College of Medicine, 199 Tun Hua N. Rd., Taipei, Taiwan; 2 Department of Medical Education, Chang Gung Memorial Hospital, Chang Gung University College of Medicine, Taipei, Taiwan; 3 Department of Thoracic Medicine, Chang Gung Memorial Hospital, Chang Gung University College of Medicine, Taipei, Taiwan; 4 Department of Nephrology, Chang Gung Memorial Hospital, Chang Gung University College of Medicine, Taipei, Taiwan; 5 Department of General Medicine, Chang Gung Memorial Hospital, Chang Gung University College of Medicine, Taipei, Taiwan; 6 Department of Medical Humanities and Social Sciences, Chang Gung University College of Medicine, Taipei, Taiwan

**Keywords:** Narrative medicine, Professionalism, Medical trainees, Qualitative study, Thematic analysis

## Abstract

**Background:**

Narrative medicine (NM) is an approach involving narrative skills and is regarded as a model for medical humanism and effective medical practice. This study aims to explore how NM impacts medical trainees’ learning of professionalism during a clerkship in a Taiwanese clinical setting.

**Methods:**

A qualitative interview study adopting a purposive sampling method was undertaken. Thirty medical trainees participated in this study, including five fifth-year medical students (MSs), ten sixth-year MSs, nine seventh-year MSs, and six postgraduate year (PGY) trainees. Thematic framework analysis was applied, and a modified realist evaluation approach was further used to analyse the interview data.

**Results:**

We identified self-exploration, reflection, and awareness of professional identity as mechanisms explaining how NM impacted professionalism learning in our participants. Furthermore, empathy, communication, doctor-patient relationship and understanding patients were identified as the outcomes of the NM intervention for trainees’ learning of professionalism.

**Conclusions:**

NM facilitates medical trainees’ self-exploration, reflection, and awareness of professional identity, thereby affecting their learning of professionalism in clinical settings. Adopting NM as an educational intervention in undergraduate medical education could play an important role in professionalism learning, as trainees can thereby be supported to gradually develop self-exploration and reflection capabilities and heightened awareness of professional identity reflectively through a narrative process.

**Supplementary Information:**

The online version contains supplementary material available at 10.1186/s12909-021-02823-4.

## Background

Narrative theory is based on the principle that people are inherently storytellers, storytelling being one of the oldest and most common forms of communication. People enter their social world narratively, making decisions and taking action within this narrative framework [1]. Rita Charon first used the term “narrative medicine (NM)” in 2000 to refer to clinical practice with narrative competency; NM provides a model for medical humanities and effective clinical practice [2]. NM promotes the interpersonal relationship between physicians and patients and aims to help doctors identify, explain and act on other people’s problems [3]. Narrative concepts, including the examination and understanding of medical reasoning, clinical relationships, empathy and medical ethics, can be used as a framework for clinical practice and ideal care while providing a means to acquire competence [4]. By narrowing the professional and personal distance among patients, physicians, their medical colleagues, and society, NM seeks to provide new opportunities for respectful, empathetic and nurturing medical care [5–8].

The US Accreditation Council for Graduate Medical Education (ACGME) includes professionalism as one of the six core competencies for physicians, with reflective learning being the key to professional development (http://www.acgme.org). Professionalism has been defined as a group of attitudes, values, behaviours and interactions that serve as the basis of the health professional’s contract with society [9, 10]. However, there are challenges in teaching professionalism, since both professionalism and reflective competence can be elusive and challenging parameters to define or measure within traditional instructional and assessment paradigms [11]. Furthermore, the field has moved from thinking about professionalism as *something we do* to considering it as *someone we are* [12, 13]. This means that professionalism is somehow embodied [14]. Thus, the allocation and use of a means through which trainees can develop their professional identity seem heavy tasks [10].

NM is considered to be an innovative and effective way to stimulate the professional development of medical students by educating them to approach the patient’s experience of illness with more understanding, compassion, reflection and empathy [15, 16]. Our previous research found that exposure to an NM program increased empathy scores among health care practitioners, as measured via the Jefferson Scale of Empathy, Health Professions Version [8]. NM competency also has the potential to strengthen communication about advance care planning (ACP) [17]. Even in surgical resident education, a narrative-based approach enhanced both practice and professionalism without being time intensive [18]. However, how narrative medicine impacts learning professionalism is under-investigated.

We aim to explore how NM can impact medical trainees’ learning of professionalism in clinical settings by asking the following research questions:

1. What are the outcomes of NM with respect to medical trainees’ learning of professionalism?
2. How does NM work to influence medical trainees’ learning of professionalism?

## Methods

### Study context

This study was conducted in Linkou Chang Gung Memorial Hospital, the largest medical centre in Taiwan. The NM model was introduced into the clerkship program (5th year) in the Department of Internal Medicine. One of the purposes of this program was to encourage teachers and trainees in fulfilling the aims of medical humanities education. Two indispensable elements of facilitating this process are mutual trust between trainer and trainee and time for trainees to reflect on their clinical practice. Using a narrative approach, clerks were encouraged to write about their daily clinical encounters with patients, their experiences of their struggles and their accomplishments shielded from the critical eye of preceptors, attending physicians or their seniors.

The NM model comprised a series of activities, described as follows:

#### Activity 1

An introductory lecture on narrative writing was held 2 weeks prior to the beginning of the semester. Here, narrative medicine theory was explained, and the process of narrative writing was introduced.

#### Activity 2

A narrative medicine workshop for clinical teachers was undertaken that included a writing assessment framework called BEGAN (The Brown Educational Guide to the Analysis of Narrative) [19]. This was led by a team leader with experience in feedback sharing. This was organised twice each year.

#### Activity 3

In the session, participants were allowed to present clinical stories from their narrative writing assignments in a variety of ways, including storytelling or poetry reading. This activity was designed to enhance the trainees’ humanistic sensitivity (i.e., the strength of their physical or emotional reactions to humanistic expression) by enabling them to recognise, interpret and be moved to action by the problems of others. Through the act of narrative writing, they were encouraged to review their journey through their clerkship and to rethink and reflect on the stories they gathered from patients. One of the objectives of this activity was to give them an opportunity to transfer their cognitive knowledge and attitudes regarding physician-patient communication into practice, with the aim of facilitating a better understanding of patients and the patient journey.

#### Activity 4

This comprised a small group discussion between 6 and 8 participants and one clinical teacher as the facilitator. In this one-hour activity, each participant read his/her narrative writing assignment, reflected on the experiences of their patient encounter and received feedback from their peers and the facilitator. The performances were assessed by the modified REFLECT (Reflection Evaluation For Learners’ Enhanced Competencies Tool) rubric [20], translated into Chinese.

### Study design

We used a purposively stratified sampling technique, ensuring the inclusion of participants from each trainee level. The sample size was determined via theoretical saturation: we continued to recruit new participants until no new themes were identified. The research project was approved by the Institutional Review Board (Chang Gung Medical Foundation Institutional Review Board, CGMF-IRB) with certification of approval (103-7363B, 104-7847C, and 105-6199C). The research process was also regulated and supervised by the CGMF-IRB. All methods were performed in accordance with the relevant guidelines and regulations.

### Participants

A total of 30 participants were recruited, including 18 males (60%) and 12 females. Five of them were fifth-year students (60% male), 10 of them were sixth-year students (60% male), 9 of them were seventh-year students (56% male), and 6 of them were postgraduate year trainees (PGYs) (67% male). The participants ranged in age from 22 to 30. We enrolled participants who had ever attended the narrative medicine intervention. Therefore, the intervention was the same as the NM model introduced in the clerkship program (5th year) in the Department of Internal Medicine.

### Data collection

We used a semi-structured one-to-one narrative interview design (Supplementary file). Informed consent was obtained face to face from all participants in the interview room. The consent we obtained from study participants was written. Participants were informed of their right to withdraw their consent at any stage of the study without penalty. Each interview lasted approximately 1 h and was conducted by 2 research assistants (YYO, CYS) in Mandarin so that participants could freely elaborate on their experiences. The relationship of the interviewers with the interviewees was independent during the interviews, with no power asymmetry. Because the research assistants were not involved in any academic or professional activities with the students, negative effects on trainees were minimized. Participants received a small monetary reward (500 New Taiwan dollars) as reimbursement. All interviews were audio-recorded, transcribed verbatim and anonymized.

### Semi-structured interview questions for medical trainees

1. Please describe your personal experiences regarding professionalism.
  1. How do you define professionalism?
  2. Give an example of when, where, and how you show professionalism in your practice.
  3. Does professionalism play a role in your clinical practice?
2. Considering your learning experiences in the NM course,
  1. Do you think that professionalism plays a role in NM?
  2. How do you learn or practice professionalism in NM? Provide specifics by recounting what took place in an actual learning session.
  3. Did you experience any obstacles in learning professionalism in NM? If yes, please describe the obstacle/s and how you dealt with them.
  4. What supports do you think you need to learn professionalism?

### Data analysis

The data were analysed inductively by 4 researchers, including the 2 investigators serving as physician educators (CDH, LKC) and the 2 research assistants (YYO, CYS). Thematic framework analysis was applied to explore how participants perceived themselves and the community they worked with (i.e., patients and the other healthcare professionals). All researchers began by analysing the same transcripts to develop the initial coding framework. Following this, one researcher (YYO) repeatedly read the transcripts individually and undertook data coding. All themes were discussed with the wider team of researchers (including the co-authors) regularly, who then gave feedback and assisted in developing the coding framework. After completing the initial coding of all the transcripts, the team as a whole then identified and clarified the main themes (and sub-themes) within the data. For the purpose of this study, we then adopted a modified realist evaluation approach to elucidate which mechanisms (often hidden psychological processes) are likely to operate in a narrative medicine intervention and what outcomes are subsequently likely to occur [21]. The study was conducted in Mandarin during the interviews and data analysis, and the English translation was performed during the period of manuscript drafting.

## Results

### Mechanisms of the impact of narrative medicine on learning outcomes for professionalism

#### Theme 1, self exploration: “*You write about yourself”*

*I think it’s a process of writing about yourself. It helps you think of the patient, which can, can help you think about the patient more consciously. From a realistic perspective, it’s like planting the memory, which makes you think about all the interactions you had with that patient. And then, I think somehow it’s helpful (C7B*)*.*

#### Theme 2, reflection: “*You turn the clinical situation into a story”*

*[You] just turn the clinical situation into a story, in which you stay away from your perspective and you observe the parts that are reasonable and worthy of discussion (C6D). I think the purpose of this* (NM) *curriculum is to change our thinking* (to understand) *that we are not superior. We need to understand patients’ thinking from a different perspective, and we need to have more practice. Yeah. In this way, we can help the patients communicate… (C6B).*

#### Theme 3, awareness of professional identity: “*Don’t let the garden be destroyed because of the temporary storm”*

*[When you] face a difficult situation or difficult person, sometimes your responses and emotions might be affected. At that time, I thought humanistic medicine is like a small garden in your heart, and this unpleasant situation or another, the bad attitude or a difficult family are like the storms trying to smash your garden. And humanistic medicine is like, like the sun, or something else. It reminds you that you still have to … you still have to keep some basics in the medical field, which is your original intention. You are responsible to these people. (…) sometimes they would jump out and shed light on the garden, reminding you to not let the garden be destroyed because of the temporary storm (7D).*

*But values cannot be changed overnight. Values are changed in people’s sub-consciousness. This activity* (i.e., narrative medicine) *basically comes with medical humanities. I don’t think it’s possible to change your patients, so I think what we can change is from the side of medical staff or so that medical staff shows their kindness. If* (you) *want to build a good relationship, there must be one side showing its kindness first. I think what narrative medicine is about is to change the doctor’s values (6B).*

### Learning outcomes of narrative medicine for professionalism

#### Theme 4, empathy

*Speaking of empathy, I think this is a matter of medicine… the training which is given in medical humanities is more like, um, probably the person is empathetic in nature. When he is taking the curriculum, he would feel he can relate to it* (to empathy) *(5A).*

*Well, because in the process of performing narrative medicine, we have to chat with patients and family members in a very effective way. We must know the patient very well. If we want to understand him, we must use a lot of empathy. It's like a skill or a way that it can break through people's heart. So that it makes strangers want to talk to you, that kind of feeling. (8B)*

#### Theme 5, communication

*Medicine is about communication among people. It’s not like a repair shop on the street, although what we are doing is more or less like fixing stuff. But what we fix are human bodies. So we have to consider their feelings. We cannot do whatever we like. We have to obtain his* (the patient’s) *consent because sometimes he would also feel not well, and we also have to take care of his feelings (8F).*

#### Theme 6, doctor-patient relationship

*I mean when you have the other [patient], you would feel familiar with the patient’s illness. I think it’s practical. And another thing is that you can think about the interaction process you have with the patient, which is the way you build a relationship (C7B*).

*‘Cos now there are many medical disputes, and many of them are due to bad doctor-patient relationships. And this* (NM course) *enables us to help patients and restore relationships with them. In this way, medical disputes might be reduced (C6B).*

#### Theme 7, understanding patients

*Actually, I think narrative medicine has an advantage, which is you can understand the patient’s medical history and diseases better, because usually we will do the medical interviews in narrative medicine (C7B). [So] I can better understand why he made the choice at that time, because I had a patient who lost his job due to his illness. At that time [I] asked him why he was not working or something else. And you learn that actually this illness had become a burden in his life, and after that, when you are delivering health education to the patients, you can be more understanding of their situation.* (You can) *be understanding of their worries or pains (C7A).*

*So suppose you were just on duty, and you just met the parents during that time, you would think they were crazy. It was just pneumonia, unnecessary to be that neurotic. But when you tried, tried to understand more details, you would gradually realize the underlying family issues (8A).*

## Discussion

We identified eight themes and categorized them as the mechanisms of the impact of narrative medicine and the outcomes of narrative medicine in terms of the professionalism of medical trainees. We categorized “self-exploration”, “reflection”, and “awareness of professional identity” as the mechanisms and categorized “empathy”, “communication”, “doctor-patient relationship”, and “understanding patients” as the outcomes of the narrative medicine intervention. The NM intervention enhanced medical trainees’ observation of and interaction with their patients and facilitated a process of self-exploration, reflection, and awareness of professional identity, thereby affecting learning outcomes for professionalism within the clinical environment. Promoting NM learning within undergraduate medical education can influence trainees’ professionalism learning outcomes through a reflective narrative process.

### Mechanisms of the impact of narrative medicine on professionalism learning: self-exploration, reflection, and awareness of professional identity

Self-exploration is an increasingly aware, imaginative, and enduring practice. It can be used to identify and challenge one’s assumptions and behaviours based on new information generated by exposure to new environments and the passage of time [22, 23]. By developing self-awareness through rigorous self-exploration, budding health professionals can maximize their abilities to handle complexity conscientiously, collaboratively, and constantly [23]. Medical trainees recall their interactions with patients by writing and thinking about their experiences. NM could potentially help them perform better self-exploration.

In medical education, reflection has been integrated into various training programs [22] and is regarded as an important element in medical education across disciplines [24–28]. It is considered to enhance the professional development of medical learners, including their critical thinking skills and clinical judgement [29], professionalism and humanism [30], communication skills and empathy [3, 31], and diagnostic competence [32]. During the narrative writing process, medical trainees narrated how they detached themselves from their own perspectives, telling patients’ clinical situations in stories through a reflective perspective. Through observation and reflection as well as discussion with their teacher and peers, medical trainees shared their opinions and steps on a journey toward awareness of professional identity and values. In our study, the trainees adopted the metaphors “humanistic medicine is a small garden in your heart” and “humanistic medicine is like the sun” to describe how resource-rich and helpful this field is. However, a bad attitude or a difficult family is like a storms trying to smash your garden. This reminds medical trainees to remain grounded in the medical field, which was their original aim in becoming a doctor. Our findings are compatible with previous literature on NM advocating and appraising its impact on fostering reflection in medical education [3, 5, 31, 33, 34].

The significance of our research includes a discussion of the professional identity of budding doctors and the factors that affect their professional development. The identity of a doctor is a self-representation achieved in stages over time. During these stages, the characteristics, values and norms of the medical profession are internalized, resulting in a person who thinks, acts and feels like a doctor [35]. According to Merton [36], the main goal of medical education is to ensure that every practitioner acquires the knowledge and skills necessary to practice medicine and that each physician obtains a professional identity. The principles of identity formation that have been clarified in educational psychology and other fields have recently been used to examine the process by which physicians obtain their professional identities [35]. Socialization, including socialization with patients and socialization with oneself, is the main process of developing professional identity: “the process by which a person learns to function within a particular society or group by internalizing its values and norms” [37, 38]. Socialization involves complex networks of social interaction, role models and mentors, experiential learning, and explicit and tacit knowledge acquisition. It affects learners and makes them gradually “think, act and feel like a doctor.” Gradually, medical students and trainees develop a professional self-awareness of thinking, acting and feeling with their patients and appreciate that the patient’s points of view may be different from their own. Therefore, enhancing narrative competence can improve professionalism and professional identity and guide clinical practice by helping practitioners understand their patients’ experience of illness and what they experience when caring for the patients [3, 39]. In this sense, clinical practice transcends medical knowledge, with doctors becoming socialized into mindfulness [40], self-awareness [41], self-knowledge, reflection and empathy [2].

### Outcomes of narrative medicine for professionalism learning: empathy, communication, doctor-patient relationship, and understanding patients

Empathy, one of the core components of professionalism, has been identified as an essential component of the doctor–patient relationship [8, 42]. It is the ability to understand patients’ situation, perspective, and feelings and to communicate that understanding to them [43]. It has been argued that good clinicians should empathize with patients [44, 45]. Indeed, research has found empathy to be the most important quality for being a “good physician” [8, 42, 46–48]. Furthermore, a positive relationship between physicians’ empathy and patients’ clinical outcomes has been found in the literature [49], suggesting that empathy might be associated with clinical competence and patient assistance [49]. Although the *ability* to feel empathy is innate, possession of it does not necessarily mean skill in using it. Thus, medical educators have tried to develop exercises and techniques to foster empathy among clinicians [7, 50]. Previous studies showed that narrative writing fostered empathy in PGY-1 psychiatric residents working with severely and persistently mentally ill patients [51]. In the course of our investigation, we noticed that medical trainees not only became more aware or developed reflection and empathy but also progressed in thinking about how to give the most practical assistance to their patients after NM activities. Indeed, the medical trainees preferred having on-site field practice and sharing their own experiences, such as through NM, rather than taking traditional lectures for professionalism education.

Good communication skills are an essential component of medical education. Doctors’ interpersonal and communication skills have a significant impact on patient care and correlate with improved healthcare outcomes [52, 53]. In our study, some participants stated that problems leading to medical disputes were often due to miscommunication. Through the NM activity, they learned to understand patients and to communicate clearly, improving the doctor’s advice and the doctor-patient relationship. The participants went beyond the body as A machine metaphor (“*medicine is not a repair shop*” and “*what we fix are human bodies*”) to express the concept that patients are more than machines and that both doctors and students should handle them with care. Narrative medicine offers opportunities for respectful, empathic, and nourishing medical care by bridging the separation among physicians, patients, colleagues, and society [3]. Our findings similarly showed that through NM, the doctor-patient relationship in the workplace was internalized, understood and fostered by the trainees that brough it to life.

It is widely accepted that the ability to understand the patient is essential to the establishment of good rapport and effective doctor/patient communication [54]. Through the process of writing and by continuously paying attention to their relationships with patients, the students improved and strengthened their understanding of patients. For example, if they found that they had inadequate medical knowledge and were not capable of giving good advice in response to the family’s questions, they would push themselves to acquire new knowledge. Participants would also find out more about the financial and social background of the patient’s family and their psychological status. Their clinical intervention is not only about curing patients’ illnesses; what is behind a patient also needs to be taken into consideration. Thus, NM could help medical trainees pay more attention to the implicit knowledge they acquire from patients and enable them to learn that empathy is a medium for improving communication and doctor-patient relationship efficiency.

### Study limitations

We acknowledge several limitations in our study. The research was conducted in a single medical institution in Taiwan, although the research site is the largest training institution in the country. In this study, we focused on qualitatively evaluating the global impact of NM in a clinical clerkship on medical trainees’ learning outcomes with respect to professionalism rather than addressing the impact of the different levels of clinical experience of these participants. For some of the participants (the sixth-year, seventh-year and postgraduate year trainees), the NM had taken place over 1 year before they were recruited for this study. Thus, the prolonged effect of the NM could vary among participants. A teacher-learner power relationship exists between the researchers and the study participants, and the extent to which this affects the data should be considered in future studies. Moreover, all medical trainees were compulsorily asked to complete one NM writing in this course rather than a voluntary NM writing, which could lead to bias.

## Conclusion

Narrative medicine facilitates medical trainees’ experiences of self-exploration, reflection, and awareness of professional identity, thereby affecting their outcomes in terms of learning professionalism in the clinical setting. Adopting narrative medicine as an educational intervention in undergraduate medical education could have impacts on professionalism outcomes, as trainees can thereby be supported in gradually embodying their self-exploration, reflection, and awareness of professional identity reflectively through a narrative process.

## Supplementary Information

**Additional file 1.** Semi-structured interview questions for medical trainees.

## Data Availability

The data are kept at the Chang Gung Medical Education Research Centre, Chang Gung Memorial Hospital, Chang Gung University College of Medicine, Taipei, Taiwan. Any questions or requests regarding the data can be addressed to Chien-Da Huang (cdhuang@adm.cgmh.org.tw).

## Abbreviations

NM: Narrative medicine
ACGME: Accreditation Council for Graduate Medical Education
ACP: Advance care planning
BEGAN: The Brown Educational Guide to the Analysis of Narrative
REFLECT: Reflection Evaluation For Learners’ Enhanced Competencies Tool
CGMF-IRB: Chang Gung Medical Foundation Institutional Review Board
PGY: Postgraduate year trainee
