# Supplementary material for: How does narrative medicine impact medical trainees’ learning of professionalism? A qualitative study
Source: BMC Med Educ. 2021 Jul 21;21:391. doi: 10.1186/s12909-021-02823-4 (PMC8296619; doi:10.1186/s12909-021-02823-4)
Supplement: Supplementary file 1 — Additional file 1. Semi-structured interview questions for medical trainees. [file 12909_2021_2823_MOESM1_ESM.docx]

**How does narrative medicine impact medical trainees’ learning of professionalism? A qualitative study**

Chien-Da Huang^1,2,3^, Chang-Chyi Jenq^1,2,4^, Kuo-Chen Liao^1,2,5^, Shu-Chung Lii^6^, Chi-Hsien Huang^3^, Tsai-Yu Wang^3^

Chang Gung Medical Education Research Center^1^, Department of Medical Education^2^, Thoracic Medicine^3^, Nephrology^4^, and General Medicine^5^, Chang Gung Memorial Hospital, Chang Gung University College of Medicine, Taipei, Taiwan; Department of Medical Humanities and Social Sciences^6^, Chang Gung University College of Medicine, Taipei, Taiwan.

**Running title:** Impact of narrative medicine on professionalism learning

**Semi-structured interview questions for medical trainees**

1. Please describe your personal experiences regarding professionalism.
   1. How do you define professionalism?
   2. Give an example of when, where, and how you show professionalism in your practice.
   3. Does professionalism play a role in your clinical practice?
2. Considering your learning experiences in the narrative medicine (NM) course,
   1. Do you think that professionalism plays a role in NM?
   2. How do you learn or practice professionalism in NM? Provide specifics by recounting what took place in an actual learning session.
   3. Did you experience any obstacles in learning professionalism in NM? If yes, please describe the obstacle/s and how you dealt with them.
   4. What supports do you think you need to learn professionalism?
